# Scaling up cervical cancer screening in the midst of human papillomavirus vaccination advocacy in Thailand

**DOI:** 10.1186/1472-6963-10-S1-S5

**Published:** 2010-07-02

**Authors:** Jomkwan Yothasamut, Choenkwan Putchong, Teera Sirisamutr, Yot Teerawattananon, Sripen Tantivess

**Affiliations:** 1 Health Intervention and Technology Assessment Program, Ministry of Public Health, Nonthaburi 11000, Thailand; 2 International Health Policy Program, Ministry of Public Health, Nonthaburi 11000, Thailand

## Abstract

**Background:**

Screening tests for cervical cancer are effective in reducing the disease burden. In Thailand, a Pap smear program has been implemented throughout the country for 40 years. In 2008 the Ministry of Public Health (MoPH) unexpectedly decided to scale up the coverage of free cervical cancer screening services, to meet an ambitious target. This study analyzes the processes and factors that drove this policy innovation in the area of cervical cancer control in Thailand.

**Methods:**

In-depth interviews with key policy actors and review of relevant documents were conducted in 2009. Data analysis was guided by a framework, developed on public policy models and existing literature on scaling-up health care interventions.

**Results:**

Between 2006 and 2008 international organizations and the vaccine industry advocated the introduction of Human Papillomavirus (HPV) vaccine for the primary prevention of cervical cancer. Meanwhile, a local study suggested that the vaccine was considerably less cost-effective than cervical cancer screening in the Thai context. Then, from August to December 2008, the MoPH carried out a campaign to expand the coverage of its cervical cancer screening program, targeting one million women. The study reveals that several factors were influential in focusing the attention of policymakers on strengthening the screening services. These included the high burden of cervical cancer in Thailand, the launch of the HPV vaccine onto the global and domestic markets, the country’s political instability, and the dissemination of scientific evidence regarding the appropriateness of different options for cervical cancer prevention. Influenced by the country’s political crisis, the MoPH’s campaign was devised in a very short time. In the view of the responsible health officials, the campaign was not successful and indeed, did not achieve its ambitious target.

**Conclusion:**

The Thai case study suggests that the political crisis was a crucial factor that drew the attention of policymakers to the cervical cancer problem and led the government to adopt a policy of expanding coverage of screening services. At the same time, the instability in the political system impeded the scaling up process, as it constrained the formulation and implementation of the policy in the later phase.

## Background

Cervical cancer is the second most common cancer in women worldwide [1]. In particular, it is a major cause of deaths in developing countries. The development of this cancer is associated with the infection of some strains of human papillomavirus (HPV) [2]. The disease is preventable through screening tests such as Pap smears, visual inspections with acetic acid (VIA) and HPV DNA test. While such secondary preventive approaches are notably effective in reducing the burden of cervical cancer in industrialized societies, the disease remains an important public health problem in poor settings because of the inadequate coverage and quality of screening services [3,4]. In Thailand, a campaign to enhance uptake of the government’s cervical cancer screening program was carried out over a three-month period in 2008. Difficulties were found, however, with gaining access to the large target group. This paper provides an explanation of the factors underpinning this radical shift, and other developments, in the Thai policy.  

Pap smear is a technique that gathers cells from the cervix to be examined for abnormalities. This cytology-based test is resource intensive as it requires a well-established infrastructure, including laboratories and well-trained health workers [3]. In many developing countries, the lack of personnel and necessary equipment are critical constraints to screening test provision [5]. In contrast, VIA is a naked-eye screening tool for the detection of precancerous tissue after the application of dilute acetic acid to the uterus cervix. Since 2005 this approach followed by immediate cryotherapy, the exposure of tissues to extreme cold to eliminate abnormal cells, has been recommended for use in low-resource settings where it is difficult to introduce Pap smears and other advanced technologies [3]. Apart from supply-side financial and technical limitations, multiple visits for testing, gaining access to results and subsequent treatment are crucial hurdles for effective screening, especially among people living in remote areas [6]. In some areas, inaccurate beliefs and poor knowledge of the disease among the target population are key factors impeding the coverage of cervical cancer screening [7].

Like other countries in the developing world, cervical cancer is a disease of high burden in Thailand, with an incidence of 20 to 25 per 100,000 female population during the past two decades [8]. The cancer is prevalent in middle-aged and older women, with approximately 2,600 women reported to die of cervical cancer every year. Pap smear tests for early detection of pre-cancerous cells have been available in health facilities throughout the country for 40 years, under the supervision of the Ministry of Public Health (MoPH)’s Department of Medical Services [9]. In addition, in 2000 VIA and cryotherapy were introduced in some areas. Coming under the responsibility of the MoPH’s Department of Health, this single-visit approach is, however, only available in 16 out of 76 provinces and has a history of slow expansion [10]. Both screening tests are financed by the National Health Security Office (NHSO) – the managing agency for the Universal Health Coverage (UC) plan.   

Despite the long-time availability of cervical cancer screening tests in Thailand, the services have not significantly contributed to a health impact in the country. A study suggests that coverage with Pap smears and VIA was as low as 11% and 8%, respectively, of the defined target population in 2005 [11]. With regard to Pap smear provision, quality problems such as mismatches between slides and names of clients, delays in delivering test results and loss to follow-up was evident. The lack of public awareness and knowledge of cervical cancer, as well as the negative perceptions of screening tests that involve genital organs, have been identified as problems in the demand side (Angkasuwapala quoted in [12]). Impediments on the supply side also play an important role. Firstly, policy direction concerning the introduction of Pap smears and VIA is unclear and there is poor collaboration between the two responsible departments [11]. As a consequence, health providers are confused and reluctant to choose one to implement. Secondly, a reliable and regularly updated information system related to cervical cancer screening and treatment is not in place. This information is necessary for program monitoring and evaluation, at both the national and local levels. Thirdly, poor attitudes towards VIA among medical doctors has resulted in their resistance to the use of this approach. VIA is perceived as a ‘low-grade’ approach, suitable for only the least-developed countries. Others argue that this intervention is not an appropriate alternative to Pap smears since evidence from randomised control studies indicates that the test is inaccurate and not of good quality [13].

Yet surprisingly, in mid 2008, the MoPH decided to scale up the coverage of its Pap smear and VIA services to meet an ambitious target. The main objective of this study was to assess the policy processes and related factors that drove this radical policy shift and shaped the implementation of such an unexpected initiative, as well as to examine the level of success of the policy.

## Conceptual starting points and research methods

The question of why policymakers pay attention to particular problematic issues, which in turn increases the chance for major policy shifts, has been addressed in many agenda-setting studies. Kingdon [14] suggests that a window of opportunity for policy innovation opens when the three independent streams of problem, solution and politics meet: a social phenomenon is recognised as a problematic issue, an appropriate solution to tackle such a problem is available, and there is sufficient political development that allows actors with new ideas to be involved in decision-making. Moreover, following Kingdon, the coupling of the three elements is facilitated by policy entrepreneurs and focusing events. The former are actors who put strong, persistent efforts into promoting certain policies, while the latter are rare and prominent events, such as natural disasters, accidental tragedies, and scientific discovery that enhance the perceived need for government action among policymakers.

This study focuses on the development of cervical cancer control policy in Thailand during the period January 2006 to July 2009. The study sought to understand the policy processes that led to the formulation of a plan for the rapid extension of cervical cancer screening services. It did this by exploring the processes of problem recognition, policy identification, and political mobilization. This analysis also examines the existence of policy champions and critical events, and their role in raising the issue not only of cervical cancer but also of its screening tests onto the Health Ministry policy agenda. Despite the fact that this paper analyses the introduction of a campaign with a very short lifespan, i.e. only three months, the authors consider this government action to be an initial effort to expand cervical cancer screening coverage. Such expansion could have been achieved if responsible officials had kept up the momentum of implementation beyond this initial period. Therefore, the scaling up notion, in the sense of expanding coverage of a priority health intervention [15], together with the way in which the scaling up process is managed [16] is employed as a framework for discussing the policy processes. 

The study applied qualitative approaches, including documentary review, in-depth interviews, and personal communication and observation derived from the researchers’ direct participation in the policy-related events. The documents reviewed comprise memoranda, meeting minutes, information sheets, research reports, letters and other documents produced by government agencies, private businesses, the media and non-governmental organizations (NGOs). The information obtained from these documents included the features of the cervical cancer control programs, including the performance and obstacles to the program, the cost-effectiveness of screening tests for cervical cancer and HPV vaccination in Thailand and other countries, the official positions of different organizations towards the scaling up of the screening tests and the introduction of the HPV vaccine. The information also included the evolution of the policy of expanding cervical cancer screening services in 2008. 

In-depth interviews were undertaken with five key stakeholders identified through document reviews and the use of the snowball technique. The interviewees included two senior officials involved in cancer screening programs, a senior administrator in the National Cancer Institute, a senior manager of the UC scheme, and a researcher in the Health Intervention and Technology Assessment Program (HITAP) who was involved in conducting and disseminating a study on cervical cancer prevention. An interview guide was developed based on the responsibilities and potential role of each interviewee in cervical cancer control. The information sought in the interviews comprised the major drivers of the policy to scale up the screening tests for cervical cancer, and the characteristics of the policy formulation and implementation processes including obstacles and interaction between key interests in different stages of the policy. A content analysis approach was employed to identify concepts and elements concerning the three streams of problem, policy and politics, and their integration as suggested in Kingdon’s [14] model, as well as to allow for newly-emerging themes. The analysis was verified by triangulation across the different sources.

Working in HITAP, a semi-autonomous research institute under the Health Ministry, all researchers in this study were involved in processes linked to the policy under investigation. They conducted a series of studies concerning preventive measures for cervical cancer and covered several different aspects such as economic evaluation, program performance, preferences and perceptions towards particular policy options of key stakeholders, and the effects of marketing strategies to encourage immunization against HPV in Thailand. Some researchers also participated in research dissemination and policy consultations. Such experiences allowed the researchers to gather insights into the policy development processes. At the same time, however, this close participation in the policy may challenge the researchers’ impartiality. To reduce potential bias, a meeting of policymakers, health officials, and representatives from the vaccine companies and NGOs was convened in early September 2009 to discuss and verify the preliminary research findings. The workshop confirmed the main findings of this study, and also provided additional information that helped to explain some elements in the policy under investigation.

## Results

### The development of cervical cancer prevention policy in Thailand

The effort to control cervical cancer in Thailand can be traced back to the 1960s when the Pap smear test was introduced as a clinical examination for individuals who attended hospital services such as family planning, ante- and post-natal clinics, and treatment for sexually-transmitted infections (Table 1) [9]. No data are available on the coverage of Pap smear tests in this period, since the service did not aim for disease screening at the population level. It is believed, however, that the number of women screened was very limited. It was not until 2002 that the MoPH’s Department of Medical Services established a cytology-based screening program for the entire female population aged 35 to 60 years. In 2000 the provision of VIA and cryotherapy, for screening purposes, began as a demonstration project in a small number of provinces with support from the JHPIEGO Corporation, the Royal Thai College of Obstetricians and Gynecologists (RTCOG) and the MoPH’s Department of Health [17]. From 2004 the two screening approaches were included in the benefit package for disease prevention supported by the UC scheme.   

**Table 1:** Chronological events related to cervical cancer prevention policy in Thailand

Date	Important event
1960s	Introduction of Pap smear as clinical examination in women who attended particular services in hospitals
2000	Instigation of VIA program as a pilot project for screening purposes
2002	Pap smear offered, as screening test, to entire female population between 35 and 60 years old
2004	Cervical cancer screening tests covered by national Universal Coverage scheme
2006	■ Launch of first HPV vaccine in the United States of America ■ Distribution of first WHO policy and technical document concerning HPV vaccination to policymakers and health workers
2007	■ Two HPV vaccine products licensed for use in Thailand ■ Consultations on cervical cancer control and HPV vaccination among WHO Member States in 6 regions including the South-east Asia and Western Pacific regional meeting in Thailand in April ■ A study to identify an optimal strategy for cervical cancer prevention in Thailand was conducted by 2 research institutes under the MoPH, namely IHPP and HITAP. The preliminary results were disseminated in December
2008	■ The GAVI Alliance announced it would consider future support of new and underused vaccines to fight deadly disease in developing countries which included cervical cancer ■ A Declaration made at the World Cancer Congress in Geneva in August called for action to ensure that HPV vaccines and other effective strategies to prevent cancer-causing infection were made widely available ■ Changes in Thailand’s Prime Minister in January, September and December and many cabinet reshuffles ■ IHPP-HITAP study report launched in August ■ From August to December, the MoPH introduced a campaign to extend the coverage of its cervical cancer screening service: the ‘116-Day initiative’
2009	■ Price for the two HPV vaccine products were reduced by the vaccine companies in February and April

Note: GAVI Alliance stands for Global Alliance for Vaccines and Immunization; IHPP for International Health Policy Program; and HITAP for Health Intervention and Technology Assessment Program.

The discovery of the association between HPV infection and cervical cancer in the 1980s [2] led to the development of a prophylactic vaccine which was expected to be a major breakthrough in reducing the morbidity and mortality from cervical cancer and other HPV-related diseases. In 2006, the first vaccine against this virus was approved by the United States Food and Drug Administration [18]. One year later, two HPV vaccine products were licensed in Thailand. However, the MoPH took the position that it would not provide the HPV vaccine in the national vaccination program, but instead recommended that the target population seek regular cervical cancer screening tests to prevent the disease. The Director General of the Department of Disease Control (quoted in [19]), revealed that the Ministry would not consider putting the HPV vaccination into its program even though it could prevent up to 70% of infections. The justification for this policy was that the vaccine price of 14,000-21,000 baht (400-600 USD) per 3-dose course was unaffordable within the government budget, and that the conventional methods of cervical cancer prevention were effective. As of April 2009 the vaccine had still not been included in the national immunisation programme nor had it been covered under public health benefit plans. It was, however, available in the private sector for those who could afford to pay out of pocket.

Although the MoPH considered screening tests as the most appropriate and practical strategy for cervical cancer control, it was not until 15^th^ August 2008 that it declared a policy to strengthen its screening services. The Health Minister announced at a press conference that the Ministry would scale up its Pap smear and VIA testing services to reach one million women between 35 and 60 years of age, irrespective of their health benefit or insurance plans [20]. This campaign would be carried out during the 116-day period from 12^th^ August to 5^th^ December 2008, known in Thailand as Mother’s Day and Father’s Day respectively. Such an announcement caught the policy audience, especially health officials and professionals, by surprise since there had been no previous warning of this radical policy shift. In particular, there was no consultation with health officials and experts in the design of the scaling up initiative. Moreover, the target of the campaign was considered ambitious given that the screening program had only had an average annual uptake of 790,000 clients in previous years [21].      

The following sections of this paper discuss how cervical cancer – a disease which had been neglected for decades – was recognised by the Health Minister as a crucial problem that required immediate action, and why scaling up Pap smear and VIA programs, rather than introducing the newly-emerging technology of HPV vaccine, were adopted as the policy interventions to reduce the disease morbidity and mortality in Thailand.

### Promotion of HPV vaccination: A reminder of neglected screening programs for cervical cancer

It was not long before the shift in Thailand’s cervical cancer screening program that the success in the research and development of an HPV vaccine was publicized globally. Promotion campaigns for HPV vaccination as an effective intervention to curb cervical cancer were implemented not only by the vaccine companies but also by international health agencies and NGOs. The common issues proposed by these organizations included scaling up the HPV vaccination and related program management in poor settings, financing concerns, increasing awareness of the vaccine availability and potential benefits of the vaccine [22,23]. Inevitably, however, deliberations around the new technology also touched upon the existing alternatives and the burden of the disease itself. In particular, the affordability and cost-effectiveness of HPV vaccination in comparison to those of Pap smears and VIA were the centre of debate, as the two vaccine products were sold at very high prices [24,25]. 

The World Health Organization (WHO) played a significant role in increasing the awareness of cervical cancer prevention approaches through several activities carried out by its Headquarters and regional offices during the years 2006-2008. These included the distribution of policy and technical information to policymakers and health professionals through a set of documents [1,26,27]. Consultations concerning HPV vaccines were held in all WHO regions. In April 2007 a meeting of Member States in the Western Pacific and South-East Asia regions was convened to discuss the current status of cervical cancer control programs in countries, the means to strengthen these services, whether and how HPV vaccination could be introduced, and the potential roles of WHO and other health and development partners [28]. Owing to the high costs of the vaccination, policymakers and experts from the two regions, including the Thai delegation, realized the need to *‘do the best that they can afford to do’* by strengthening the efforts for cervical cancer screening.

After the launch of the HPV vaccine on the global market, the vaccine industry became an active player in cervical cancer control policy debates. As key informants revealed in interviews in May 2009, when the vaccine became available in Thailand, representatives of the two vaccine producers contacted senior health officials in the Health Ministry and public health insurance office as well as experts and clinicians, with the aim of pursuing publicly-financed HPV vaccination as part of the national immunization program and the UC scheme. Moreover, the industry convened conferences and academic fora, occasionally in collaboration with NGOs, to discuss the feasibility of and potential impediments to HPV vaccination [29,30]. In most of the meetings, the vaccine price and its impact on the government budget were highlighted by health officials and policy researchers. Also, the strengths and weaknesses of cervical cancer screening services were occasionally mentioned by both those opposing and those supporting a publicly-subsidised HPV vaccination program. In early 2009, by which time it was clear that one of the government’s main concerns was the unaffordable cost of the vaccine, the two vaccine producers offered significant price reductions in their products. Nevertheless, by April 2009, the pricing strategy had still not succeeded in encouraging national authorities to introduce mass immunization against HPV.

The introduction of HPV vaccine and related discussions and debates can be regarded as focusing events for cervical cancer screening services. As Kingdon (1984) argues, focusing events may contribute to a policy change by triggering sufficient public demand for a policy innovation that it cannot be resisted by the authorities. An early study in Australia illustrated the critical role of public opinion and political mobilization in encouraging a government decision to adopt publicly-funded HPV vaccination [31]. Although in Thailand there was no strong public movement on the issue, debates about the HPV vaccine certainly focused attention on cervical cancer as policy problem amongst policy makers. 

### The role of policy entrepreneurs

Thai experts held different positions on whether to introduce the HPV vaccine nationwide. Some clinicians argued that the vaccine, when introduced alongside Pap smears, is the most effective means to prevent cervical cancer and other HPV-related diseases. Occasionally, these clinical experts expressed their support of the vaccination in conferences and public education events convened by NGOs and vaccine companies. However, a group of obstetricians and gynaecologists emphasized the need to enhance screening test provision since the cost of HPV vaccination was unaffordable [10]. In addition, the RTCOG and oncologists associations issued a joint statement in July 2008 titled ‘HPV vaccines and cervical cancer prevention’ [32] aimed at providing the public with guidance for encouraging the rational use of HPV vaccines.

In the face of these different positions, it was policy researchers from two institutes under the MoPH who had a crucial role in informing policy decisions with evidence. From early 2007 to 2008, the IHPP and HITAP, in collaboration with experts from the RTCOG and other organisations, carried out a project: ‘Research for the development of an optimal policy strategy for prevention and control of cervical cancer in Thailand’ [11], with financial support from the World Bank’s Population and Reproductive Health Capacity Building Program. The study provided insights into the inadequate performance of cervical cancer screening services and proposed an optimal strategy to control the disease. The findings illustrated substantial room for improvement in the coverage and quality of the national cervical cancer prevention program. Furthermore, the study suggested that screening tests are cost-saving, in comparison to treatment of cervical cancer, and that the most cost-effective option to prevent the disease is the combination of VIA and Pap smears, by providing VIA every 5 years to women 30 to 45 years of age, and Pap smears every 5 years to those between 46 and 60 years old. Importantly, HPV vaccination was found to be cost-ineffective owing to the very high price of the vaccine.

In December 2007, the preliminary results of two elements of this study, the performance assessment of the national cervical cancer screening program and the economic evaluation of policy options for the prevention and control of cervical cancer in Thailand, were discussed amongst representatives of MoPH departments, the NHSO, the RTCOG and the vaccine companies. It was the first time these stakeholders obtained comprehensive information concerning cervical cancer control strategies. One of the researchers in this project pointed out in an interview in early 2009 that she and her colleagues were often invited to give presentations on the study results at policy fora including those organised by the Department of Health’s Reproductive Health Division, the Subcommittee for the Development of Benefits and Service System under the UC, and the NHSO’s Working Group on the Development of Cervical Cancer Screening System. Thus, policymakers who were responsible for priority setting and resource allocation in the health sector were exposed to information on the performance of the existing cervical cancer prevention services and also the economic aspects of screening tests and the new technology, the HPV vaccine. Furthermore, HITAP disseminated the study findings and information concerning the rational use of the HPV vaccination to the public through different channels such as a public forum in August 2008 and television programmes. 

### The broader political context

After a military coup in 2006, Thai politics were in a chaotic and unstable state. A series of street demonstrations were held seeking to achieve the removal of the administration, which had been elected in January 2008, and calling for a new election. However, the coalition government did not dissolve the parliament, but rather appointed new Prime Ministers and Cabinets. During 2008 there were 4 prime ministers and many cabinet reshuffles. In response to the increase in social disorder in mid-2008, several public programs were introduced with the aim of gaining public support and extending the political life of Cabinet members. The one hundred and sixteen day period from 12^th^ August to 5^th^ December was selected by the Cabinet as the timeframe for implementing populist policies by all government ministries. The MoPH’s 116-Day campaign focussed on the extension of cervical cancer screening services. A senior advisor in the MoPH who often worked closely with the Health Minister noted that, *‘I informed the Minister that if he pursued this policy, he would win the heart of all women. Then, he agreed and gave me the green light.*’ In the same vein, another senior official argued in an interview in 2009 that, *‘I discussed with the Minister and informed him that the screening program including essential infrastructure for the service scaling up already existed, but a clear policy was needed. If the Minister pushed it, it could be successful and he would gain social credit without any significant additional investment.’*

Prior to the political crisis of mid-2008 the Health Minister might neither have recognised the launch of the HPV vaccine on the global and domestic market, nor obtained information on the vaccine, or even existing cervical cancer control programs. However, such developments posed an important question to health officials, experts and researchers: was HPV appropriate and feasible in the Thai setting? The IHPP-HITAP study was, therefore, timely and addressed the demand for evidence-based recommendations. In interviews, senior health officials argued that the study findings were not surprising and confirmed their belief that HPV vaccination was not suitable in Thailand unless the vaccine price fell significantly. As these key informants indicated, the recommendation to strengthen the national screening programs was largely in line with what they had already recognised as a priority action, even though some of them felt that the combined introduction of VIA and sequential Pap smears was not necessary.

Interview respondents asserted that dissemination of the IHPP-HITAP study was influential in prompting them to call for the improvement of the national cervical cancer screening services. As a high ranking official in the MoPH pointed out in an interview, *‘If you ask whether my participation in the seminar prompted me to raise this issue to the Health Minister, I can say that it played a part. It was the IHPP-HITAP study that made me increasingly interested in the issues of HPV vaccination and cervical cancer screening.’* A similar argument was made by the Director General of the Department of Health who noted that he recognised the results of the economic evaluation and felt the need to convey the information to the Health Minister, as he was among the few persons who could play a leading role in improving the fragmented screening program. As these officials put it, they made the proposal to this key policymaker because they were aware that the cabinet was keen to introduce any programs that offer benefits to the general population, and felt that strengthening the national cervical cancer screening services would meet this political demand.

### What was the scaling up process?

In interviews in 2009, two senior health officials noted that they proposed the scaling-up of cervical cancer screening to the Health Minister in early August 2008. Unexpectedly, in a press conference on 15^th^ August, the Health Minister announced the 116-Day campaign. This suggests that the policy was adopted and formulated in a very short period of time. As highlighted by the respective program managers, neither the operational plan, nor the mechanisms for monitoring and evaluation of the service extension, were put in place. Moreover, no participation from stakeholders such as clinical experts and health providers could be observed during the policy formulation and implementation process. Financial resources from the MoPH and the NHSO were, however, allocated to public information activities.

An administrator at the National Cancer Institute – the main agency responsible for the 116-Day initiative – pointed out that although this policy aimed to strengthen screening services for cervical cancer, there was no guidance regarding revision of the operating procedures for screening practices at health centres and community hospitals where the majority of the tests were provided. In addition, no solutions to counter the already known obstacles to the provision of screening services were identified; these included the inadequate number of cytologists, mistrust in the VIA approach among physicians, problems related to the referral system between primary care settings and higher-level institutions. During the implementation phase of this campaign sporadic media coverage and public-information events could be observed. A high-ranking official who claimed a role in raising the cervical cancer screening issue with the Health Minister revealed that this initiative was not introduced in the way he had expected: ‘*I do not expect to see the policy for expansion of cervical cancer screening being implemented like this, I wished to see it implemented in a more rigorous and sustainable way*.’

Evidence indicating the achievements of the campaign is provided by the number of clients served. On National Cancer Day, 9^th^ December 2008, the Director of the National Cancer Institute reported through the public media that 82,700 women were screened for cervical cancer during the 116-day period [33]. This figure was only slightly higher than the number of screening tests performed during the same period in the previous year. Although there was no formal evaluation of the 116-Day initiative, key informants in this study shared a common view that the policy scale up was unsuccessful. They identified the barriers to scaling up as: lack of policy clarity on the introduction of VIA and its harmonisation with the long existing Pap smear program, inadequate preparation for the 116-Day campaign, and a low awareness of the need for the service in the target population.

It is noteworthy that in conjunction with the 116-Day campaign, there was also an initiative to strengthen the screening program for cervical cancer in Thailand. Despite being mandated as a health care purchaser, in October 2008 the NHSO appointed a task group to the Development of the Cervical Cancer Screening Service System. This group comprised officials responsible for the Disease Prevention and Health Promotion Fund, representatives from the Department of Health, the Department of Medical Services and the RTCOG, and researchers who were involved in the World Bank funded study. In a meeting on 20^th^ November, 2008 the task group endorsed the use of either Pap smears or VIA for cervical cancer screening and made it clear that either of the tests could be reimbursed under the UC plan. This meant health providers could choose the approach they considered to be the most appropriate to their setting. For instance, VIA would be employed in those facilities unable to provide the Pap smear service and proper treatment of abnormal cells. Recognizing the inadequate performance of the screening test service, the task group also called for proposals from interested health agencies to extend the service. This was to meet the target of screening 80% of women aged 35 to 60 years old within the next five years. However, this effort had only made limited progress when the 116-Day campaign ended in December of the same year.

## Discussion

This Thai case study illustrates a number of critical factors in the problem, policy and political streams that encouraged the rise of cervical cancer screening onto the governmental agenda – the process that led to authoritative decisions and eventually, the 116-Day initiative in 2008. In line with Kingdon’s [14] agenda setting model, we recognise the discovery and distribution of the HPV vaccine as a focusing event – an obvious, sudden, relatively uncommon event that highlights a problematic issue and/or a solution to which policy authorities and the public pay attention. Without this discovery and its distribution, policymakers and other key policy stakeholders in Thailand, and around the world, might not have recognised the cervical cancer burden and available prevention measures. In particular, since the newly-introduced technology was unaffordable for poor countries, screening tests were revisited and discussed by international and national health organizations, as effective alternative means of curbing the disease. Health authorities and experts in most settings did not recommend HPV vaccination as a substitute for screening services, but as a complementary measure. 

Policy researchers in the Health Ministry played a significant role both in generating evidence concerning the cost-effectiveness of cervical cancer screening services and HPV vaccination, and in disseminating the research findings to policymakers and administrators in responsible agencies. Such actions by these policy entrepreneurs enabled the coming together of the problem and policy streams. These actions did this by getting the leaders of national health authorities to recognize the magnitude of cervical cancer burdens and become aware of the room for improvement in the screening program. All this happened while the country was faced with a political crisis in which members of the Cabinet needed to present the public with their successful policies in order to gain the popularity that would help secure their government’s position. It is noteworthy that despite catching the attention of researchers, health officials and politicians, the issue of cervical cancer and screening tests did not reach the attention of the general public, which means that most Thai people, including those targeted by the services, were not involved in the policy shift. Indeed, the analysis also illustrates that the policy shift was influenced by a limited number of actors based solely in the MoPH, with other interests, such as the media and NGOs, playing no role.

Although agenda setting generally involves the rise of interest in a policy problem and, in some instances, its corresponding solutions, this process does not guarantee success in subsequent stages of policy formulation and implementation [34,35]. The political rhetoric and top down instructions associated with the 116-Day initiative were inadequate to achieve the extended target for cervical cancer screening. Following Hanson et al [36], the scaling up of essential health services requires several elements, some of which did not exist for this initiative. These included, in particular, those defined as strategic management factors such as involvement and collaboration between key actors in decision making and implementation units, as well as decentralized mechanisms for policy formulation, planning and administration. Hanson et al. also argue that the constraints to extending public health program coverage may involve contextual factors such as political instability and weak governance. Key informants in this study certainly maintained that the shortfalls in the 116-Day initiative resulted mainly from the chaotic political situation as this did not allow sufficient preparation and engagement between the central departments and local service providers.

The findings of this analysis are in line with existing literature which highlights the crucial role of political factors, including policy elites, not only in facilitating and impeding policy shifts but also in guiding the success or failure of the innovations in particular settings [37,38]. An analysis of health financing policy in South Africa and Zambia demonstrates, meanwhile, the undesirable consequences of rapid policy changes underpinned by political drivers, as they may fail to carry forward through policy formulation and implementation [39]. These African experiences also emphasize the general importance of management strategies in successfully translating policy prescriptions into action [16]. These include the needs for relevant technical capacity, effective design of proposals and clear communication of policy changes. In the scaling-up of cervical cancer screening services in Thailand, although the capacity to conduct technical analysis existed in the Health Ministry, research institutes and even in peripheral health facilities, this expertise was not consulted in devising the ambitious campaign.

An important limitation of this research is that it does not explore the process of scaling-up cervical cancer screening at the peripheral level. Therefore, the real problems faced by health providers in implementing the MoPH’s 116-Day campaign to strengthen the cervical cancer screening services have not been identified. It is also worth noting that official statistics indicate little extension of services during the program implementation period. However, given that a top-down model of policy implementation was applied, it is not surprising that this initiative was not successful. An extraordinarily short period was available to develop this national-scale campaign protocol and lessons learned from past experience related to the screening program were not drawn on as inputs in formulation of the policy. Without action to counter the already known obstacles to service provision, it was clearly difficult to meet the campaign’s ambitious target.   

Fortunately, though, the current development of Thailand’s cervical cancer screening program indicates subsequent improvement regarding its coverage, effectiveness and efficiency. The continuing advocacy for HPV vaccination provides a conducive context for the government to strengthen the screening service provision as it is the only affordable choice currently available. Furthermore, from early 2009 the MoPH’s Department of Health, in collaboration with other organizations, including the Department of Medical Services, has been introducing a pilot program which offers cervical cancer screening, with the operational strategy as recommended by the IHPP-HITAP study, in a number of provinces. Meanwhile, the NHSO and the MoPH are implementing a plan to enhance existing databases and reporting systems related to cervical cancer control. All of these actions may, to some extent, help to ensure better performance of the screening program which will be gradually scaled up in the future.

It is often argued that the main obstacles to health system development in resource-limited settings are inadequate financial and human resources, as well as the lack of relevant and reliable evidence to guide proper policy decisions. This Thai case study shows that even though scientific evidence concerning the safety, efficacy, effectiveness and value for money of policy options for prevention and control of cervical cancer were available, with political will, adequate financial support and a well-established infrastructure, the scaling up of the screening service in Thailand did not achieve its promise in its explicit phase of implementation. This suggests the need for policy managers to have a better understanding of the processes of policy development, including insight into the roles of stakeholders, their interests, and interactions with the health system context. However, analysis of health policy is rarely recognized and applied in developing countries’ academic institutes and health administrative authorities [40].

## Conclusions

In conclusion, this study provides an illustration of the effort to strengthen cervical cancer prevention programs in a middle-income country, with relatively strong health care delivery and financing systems. This policy innovation took place within the Health Ministry just after the HPV vaccine was launched onto the market and advocated for by interest groups including international organizations, the vaccine industry and private health providers. However, the scaling-up of the screening services did not reach its ambitious target inside its own timeframes as it was largely driven by political factors and was not well devised.

## Competing interests

The authors declare that they have no competing interests.

## Authors’ contributions

JY contributed to the conceptualization and study design, and oversaw all aspects of data collection and analysis. CP planned, conducted and analysed the interviews. TS contributed to the document reviews, data analysis and coordination of the study. YT and ST were involved in drafting and revising the manuscript. All authors have given final approval of this manuscript.
